# Milk as a Source of Infection

**Published:** 1897-05

**Authors:** G. M. Corput

**Affiliations:** Atlanta, Ga.


### MILK AS A SOURCE OF INFECTION.

BY G. M. CORPUT, M. D., ATLANTA, GA.

While the subject of the possibility of infection by contaminated water is recieving such universal attention from both the profession and the laity, and articles, for and against, from many pens, are constantly appearing in both the secular and medical press, I would like to call attention to another source of infection, which is not receiving, especially from the profession in the South, the attention which its gravity demands. I refer to the milk supply of our cities.

That the danger from this source is real and serious, has been clearly shown by the observations of such careful experimenters as Bollinger, in Germany; Ernst, in Boston; Surgeon-General Sternberg, and many others.

The degree to which contamination takes place under ordinary circumstances may be judged from the investigations of Sedgwick and Batchelder, in Boston, in 1892. In fifteen specimens of milk handled in the usual way, and examined a few hours after it was drawn, the average number of bacteria found in each cubic centimeter was 69,143. The principal source of this contamination was undoubtedly from unclean cows, attendants, utensils, stables, etc.

No doubt a great proportion of these germs are harmless, but with them are others, which, if not strictly pathogenic, hasten fermentative changes in the milk and greatly interfere with its digestibility.

It is a fact that milk from a cow during her "bulling period" may, and frequently does cause severe attacks of indigestion in infants who get their nourishment from such a source, thus showing that the milk, at this time, contains some form of toxic products.

The question presents itself, if milk from cows is so dangerous, how is it that we have used it for years with impunity? The reply must be, that it may not have been so innocuous as we have imagined.

It is only within recent years that the profession has come to regard milk as an important factor in the etiology of tuberculosis.

Infection from this source has been proven, and the bacilli found in milk by such careful observers as Gerlach, Bang, Ernst, and many others.

Striking illustrations of this are sometimes afforded in the lower animals. Hirshberger reports the interesting fact that that an owner of a herd known to be tubercular withdrew the milk from market, and used it, without boiling, to fatten his hogs. These animals, almost without exception, developed the disease and had to be slaughtered. There is no reason for believing that young children, or even adults, are less susceptible to infection in this manner than the lower animals. The great frequency of intestinal and mesenteric tuberculosis in children no doubt finds here its explanation.

In 119 autopsies, personally conducted by Prof. L. Emmet Holt, in children under five years of age, tubercular lesions were found in either the intestines or mesentery in seventy-eight cases.

In 127 autopsies of fatal tuberculosis in children, noted by Woodhead, 100 were found to have tubercular lesions in the mesentery.

Dr. Shakespere, of Philadelphia, states that "fully one-fifth of all the deaths of infants and children, feeding on ordinary milk, are due to tuberculosis commencing in some part of the digestive tract."

Dr. Hamaker, of Meadville, Pa., reports that, since tuberculous cows have been eliminated from the herds supplying that city with milk, there is much less disturbance of digestion of children than formerly, and that with a population of 12,000, last season there were only two deaths from "cholera infantum."

Leonhardt reports the case of a healthy infant, of healthy parents, which was weaned and put on cow's milk. The child died of tuberculosis of the meninges, intestines and mesentery. The cow which gave the milk was killed and found to be tubercular. Another child, fed from the milk of the same cow, died of tuberculosis about the same time.

Sontag reports the case of a six months' infant, of healthy parents, which at autopsy showed tubercular lesions; investigation proved that it was fed with milk from a tubercular cow.

Demme reports the case of a four months' infant, which at autopsy showed tubercular lesions of the mesenteric glands. There was no tuberculosis in the family for two generations on either side. The milk on which it was fed came from a cow with general tuberculosis.

Bollinger cites the case of a boy of five years, who sickened with ascites and enlarged glands in the abdomen. At autopsy the chief lesion was tuberculosis of the abdominal lymphatics.

There was no tuberculosis in the family, but the boy was in the habit of drinking warm milk from a cow, which growing thin before the boy died, was killed, and found to be tubercular.

It is unnecessary to further multiply the citations of the results from the experiemntal investigation of the question under discussion, or the results of clinical and pathologic observations as to the direct transmission of the disease through the use of tuberculous milk.

The phase of the subject which gives rise to differences of opinion among the profession to-day is not whether the milk from tubercular cows is dangerous, but at what stage of the disease is it dangerous, and how widespread is the disease.

Authorities differ as to the extent to which tuberculosis prevails amongst domestic animals, it has been variously estimated that from ten to twenty-six per cent. of milk cows of various breeds are tubercular.

Locality has much to do with this, however, the disease being more prevalent when cattle are kept closely confined during the greater part of the time. The Jersey and Guernsey cattle seem more prone to the disease than other breeds.

The danger of infection is, of course, greater when the disease has become general than when it is localized, and is especially great when the udder becomes diseased, but that there is danger at all stages is shown by the following notes of Dr. Ernst, the Bacteriologist at Harvard, in a report made by him to the Massachusetts Society of Agriculture in 1894.

The desire of the society was to determine whether or not the milk derived from tubercular cows might contain the infectious material of the disease, and in this way become dangerous when used as an article of food, and this question was of necessity to be divided into two parts; first, whether this infection of the milk existed in cases in which there was actual disease of the udder, and second, whether it might exist in cases in which the udder was apparently, or actually healthy, the disease existing in other parts of the body. In regard to the first part of the question, plain common-sense showed that the danger of infection was a real one, and besides there existed at the time sufficient experimental data to prove the fact, so that there was very little dispute that under such circumstances milk should not be used for food, certainly not in an uncooked condition. Evidence since then, in the same direction, has constantly accumulated, and now there are few to challenge the opinion that milk from cows with tubercular udders should be condemned as food.

Upon the second point, however, as to whether the milk from cows with tuberculosis, but not of the udder, might be dangerous, there was great diversity of opinion, and almost no experimental evidence upon which to base what opinion there was.

Work was prosecuted under the following headings; first, a careful and persistent microscopic examination of the milk from such cattle. Second, inoculation experiments with such milk. Third, experiments in feeding with such milk.

After two years of continuous and methodical examinations of milk from cattle at the experimental farm at "Mattapan," the following conclusions were reached:

Thirty-six cows having been examined and one hundred and twenty-one examinations of milk and cream made, each examination was made from bottom and top fluid. In every case at least a dozen cover-glasses were used for each examination, and fifteen minutes time was spent over each cover-glass The staining was invariably "Koch-Ehrlech," twenty-four hour method, the bacilli of tuberculosis were found in one or more of the cover-glasses upon nineteen different occasions; these nineteen positive results were obtained from twelve different animals, and the bacilli were found in about equal proportion in milk and cream, and were actually found in the milk of one third of the animals.

These conclusions were confirmed in twenty out of the thirty-six animals by careful post-mortem examinations.

The inoculation experiments were made on guinea pigs and rabbits. In six weeks eighty-eight guinea pigs were inoculated with milk and cream from fifteen different cows, twelve of which were tubercular. About one-third developed the disease.

Of ninety rabbits inoculated, sixteen showed tubercular lesions.

Twelve healthy pigs fed on milk from tubercular cows with healthy udders showed fifty per cent. of tuberculosis.

And of twenty-one calves similarly treated eight developed the disease.

In the twenty-five cows used for experimental purposes not one had any tuberculosis of the udder.

Of these cows, nineteen had calves which were killed when six days old without any evidence of tuberculosis, showing the absence of hereditary infection.

The summary of the report states, that among three thousand animals examined, eighteen per cent. were found diseased, and over eight per cent. of the remainder were suspicious.

From these experiments the following conclusions are drawn.

While the transmission of tuberculosis by milk is probably not the most important means by which the disease is propagated, it is something to be guarded against most carefully.

The possibility of milk from tubercular udders containing the infectious elements is undeniable.

With the evidence here presented, it is equally undeniable that milk from diseased cows, with no appreciable lesion of the udder, may, and not infrequently does, contain the bacillus of the disease.

Therefore all such milk should be condemned as food.

These deductions are legitimate, and should lead us to examine with great care all milk products.

Not only has it been proven that tuberculosis has been transmitted through milk to man, but that diseases wholly allied to man have been carried through this medium. The wonder is that it is not more so. A daily food so well calculated by constituents to be a suitable abiding place for germ life, which develops and multiplies indefinitely, requires more than ordinary attention while in the hands of the producer and tradesman.

There are many cases on record with undeniable proof of the spread of typhoid fever, diphtheria, and many other forms of enteric disease through this medium alone.

I beg to cite one prominent case which excited more than local interest, occuring in the village of Stamford, Conn., in April, 1895.

Typhoid fever began to appear during the early part of the month. No special significance was attached to its invasion, as at that time but few cases had developed, but before the end of the month 209 cases had been reported to the health officers.

In the meantime diligent search was set on foot to find by what avenue this intruding enemy was seeking the life and happiness of the community, but before it was found from whence it came, it had as its claim 386 cases and twenty-two deaths.

Every one of the cases was traced directly to a foul well in the stabling shed of H. R. Blackman, a retail milk dealer. A map was drawn showing the location of the typhoid cases and Blackman's delivery route. The Stamford doctors had not driven off the milkman's route in their visits. Every house with typhoid patients was a stopping-place for the milkman's horse.

This case shows clearly that milk should not be sold from dairies where such diseases as typhoid fever, diphtheria, tuberculosis, croup, scarlet fever, measles, smallpox, erysipelas, and syphilis, are prevalent in the human family.

There is room for much reform in the manner of managing the milk supply of our cities. I will not go into the details of the dairy, which are many and complex.

The method to be pursued and the material used are quite essential. Scrupulous cleanliness should be observed.

The milk, as soon as it is drawn from the cow, should be cooled to a temperature of 60° F. and kept in an atmosphere free from all odors and contaminating influences of whatever nature until it is ready to be taken to market. I believe that as the result of carelessness or ignorance on the part of some dairyman, premature graves have been dug for children; and the bright hope of their maturity, which was twin in the breasts of the parents at their birth, has been broken and washed away in bitter tears.

The question naturally arises, how are we to protect ourselves from this danger? The answer is plain. To guard against the dangers of infection through the use of tubercular milk, we should urge the necessity of the thorough inspection of all animals kept for dairy or breeding purposes, by a competent veterinarian, and those showing a trace of disease should be condemned. It should be the duty of this veterinarian to visit each herd twice a year, or oftener, and to test each cow with "tuberculin," when in his opinion it is necessary after the first initial test.

With these protective measures carried out in the spirit of the law, and that legislation be made for the protection of dairymen by remunerating them for the loss of their fine cattle, which are peculiarly liable, much may be done towards stamping out this dread disease.

